# Effects of Long-Term Storage at −80 °C on the Human Plasma Metabolome

**DOI:** 10.3390/metabo9050099

**Published:** 2019-05-17

**Authors:** Antje Wagner-Golbs, Sebastian Neuber, Beate Kamlage, Nicole Christiansen, Bianca Bethan, Ulrike Rennefahrt, Philipp Schatz, Lars Lind

**Affiliations:** 1 Metanomics Health GmbH, Tegeler Weg 33, 10589 Berlin, Germany; antje.wagner-golbs@metanomics-health.de (A.W.-G.); nicole.christiansen@metanomics-health.de (N.C.); bianca.bethan@metanomics-health.de (B.B.); ulrike.rennefahrt@metanomics-health.de (U.R.); philipp.schatz@astrazeneca.com (P.S.); 2 Biocrates Life Sciences AG, Eduard-Bodem-Gasse 8, 6020 Innsbruck, Austria; sebastian.neuber@biocrates.com; 3 Department of Medical Sciences, Cardiovascular Epidemiology, Uppsala University, Dag Hammarskjöldsv 10 B, Uppsala Science Park, 75237 Uppsala, Sweden; lars.lind@medsci.uu.se

**Keywords:** biomarker, long-term stability, storage, plasma, metabolomics, mass spectrometry

## Abstract

High-quality biological samples are required for the favorable outcome of research studies, and valid data sets are crucial for successful biomarker identification. Prolonged storage of biospecimens may have an artificial effect on compound levels. In order to investigate the potential effects of long-term storage on the metabolome, human ethylenediaminetetraacetic acid (EDTA) plasma samples stored for up to 16 years were analyzed by gas and liquid chromatography-tandem mass spectrometry-based metabolomics. Only 2% of 231 tested plasma metabolites were altered in the first seven years of storage. However, upon longer storage periods of up to 16 years and more time differences of few years significantly affected up to 26% of the investigated metabolites when analyzed within subject age groups. Ontology classes that were most affected included complex lipids, fatty acids, energy metabolism molecules, and amino acids. In conclusion, the human plasma metabolome is adequately stable to long-term storage at −80 °C for up to seven years but significant changes occur upon longer storage. However, other biospecimens may display different sensitivities to long-term storage. Therefore, in retrospective studies on EDTA plasma samples, analysis is best performed within the first seven years of storage.

## 1. Introduction

High-throughput metabolomics is a powerful tool for systematic metabolite profiling of complex biological systems to understand disease mechanisms and to identify novel clinical biomarkers for diagnosis, prognosis and treatment response. For example, in recent years, this technique has been successfully applied for developing biomarkers and gaining deeper knowledge of common and devastating diseases like cancer [1,2], type 2 diabetes [3,4,5] or cardiovascular diseases [6,7,8]. One of the most common types of sample matrix used in these research areas is blood-based material, such as serum or plasma, because of its minimally invasive accessibility and the extensive coverage of the human metabolome. However, since metabolites are sensitive to pathological alterations and improper sample handling, accurate quality assurance as well as quality control are mandatory to obtain reliable results and to ensure reproducibility [9,10,11,12,13,14,15,16,17,18]. The main source of laboratory uncertainty is pre-analytical variability. Failures in identifying confounders could lead to serious misinterpretations and erroneous clinical decisions [19]. Inability to complete metabolic biomarker studies due to pre-analytical confounding factors have been reported [20], and similar challenges have been observed in transcriptomics, peptidomics and proteomics research [21,22,23]. The most relevant technical issues in the pre-analytical phase are sample collection, processing, transport, and storage [16,24]. In clinical research, and particularly in -omics approaches, sample quality must be guaranteed by standard operating procedures (SOPs) to eliminate pre-analytical bias caused by inappropriate sample handling or inadequate storage conditions. However, in multicenter studies it is difficult to ensure that each institution strictly adheres to the sample preparation procedures as defined by SOPs. While specific SOPs can be determined for sample collection, processing and transport, changes in metabolite concentrations during storage are challenging to control and cannot be completely avoided. Nevertheless, the experimental design of long-term retrospective and prospective epidemiological studies often requires the use of frozen samples that were collected months, years or even decades ago, and were thus subject to different retention periods prior to analysis [25]. Therefore, knowledge of metabolite stability during long-term storage is of paramount importance to allow unbiased comparisons of samples collected at various time points and stored for different periods of time.

Cryoconservation of biospecimens in liquid nitrogen is considered the preferred method of sample preservation due to excellent sample stability [26]. As an example, ascorbic acid concentrations were reported to be significantly stable over a storage period of eleven years in liquid nitrogen tanks [27]. However, liquid nitrogen poses handling hazards and might be too expensive in biobanking environments [28], Therefore, sample storage in freezers at a temperature of at least −80 °C was recommended to maintain long-term integrity of biomarkers [29]. Criteria for selecting the optimum storage temperature were reviewed by Hubel et al. [30]. Most of the existing studies on metabolite stability during sample storage focus on short-term stability or effects of repeated freeze-thaw cycles [12,13,14,15,18,31,32,33], while the impact of long-term storage on the metabolic fingerprint is not yet fully understood. Indeed, Hustad et al. investigated the influence of storage time on biomarkers related to vitamin B metabolism in serum and plasma samples stored for up to 29 years [34], Yang et al. examined two plasma sample cohorts at two different time points within a five-year-framework [11], and Abuja et al. simulated the effect of storage time by repeated temperature changes [26], Recently, Haid et al. found that the levels of amino acids, acylcarnitines, glycerophospholipids, sphingomyelins and the sum of hexoses in plasma samples are altered after five years of storage [35]. 

In the present work, we investigated the impact of storage time on the human ethylenediaminetetraacetic acid (EDTA) plasma metabolome in samples that were stored at −80 °C for up to 16 years prior to analysis. Besides providing new basic insights into the stability of metabolites during long-term storage in the freezer, these results and the derived knowledge of differing sensitivity of metabolites to storage effects will be valuable in the development of novel biomarkers.

## 2. Results

### 2.1. Long-Term Storage Affects the Plasma Metabolome

In order to identify stable and unstable metabolites during long-term storage, we analyzed the changes in concentration over different storage periods. Due to the study design (repeated phlebotomy of subjects at ages 70, 75 and 80) storage time was correlated with subject’s age and hence our analysis focused on storage differences within each of the three subject age groups (70, 75 and 80 years of age). Table 1 and Table S1 show the changes of the metabolite levels in plasma samples after storage for up to 16 years at −80 °C, within the different subject age groups, i.e., samples stored for four up to seven years were compared to those stored for less than four years, samples stored for more than nine up to eleven years were compared to those stored for more than 7 and up to 9 years, and samples stored for more than 14 years and up to 16 years were compared to samples stored for more than eleven up to 14 years. We found that 226 out of 231 metabolites remained stable during the first seven years of storage. After storage periods of up to eleven years versus those of up to nine years, 26% of the tested metabolites were affected (20% increased, 6% decreased), and prolonged storage of up to 16 years versus those of up to 14 years resulted in a statistically significant increase of 7%, and a decrease of 4%, of the analyzed metabolites.

Though more or less all major metabolite classes were affected by long-term storage complex lipids, fatty acids, energy metabolism molecules, and amino acids and their related compounds displayed the highest sensitivity against long-term storage. In conclusion, this data indicate that the human plasma metabolome is adequately stable in the first seven years of storage but is sensitive to prolonged storage for up to 16 years. It is interesting to note, however, that the plasma metabolome showed less significant changes when comparing the two longest storage time groups as compared to the differences the storage time group of up to eleven years is compared to that of up to nine years.

### 2.2. Impact of Long-Term Storage on Selected Plasma Metabolites

**Figure 1:**
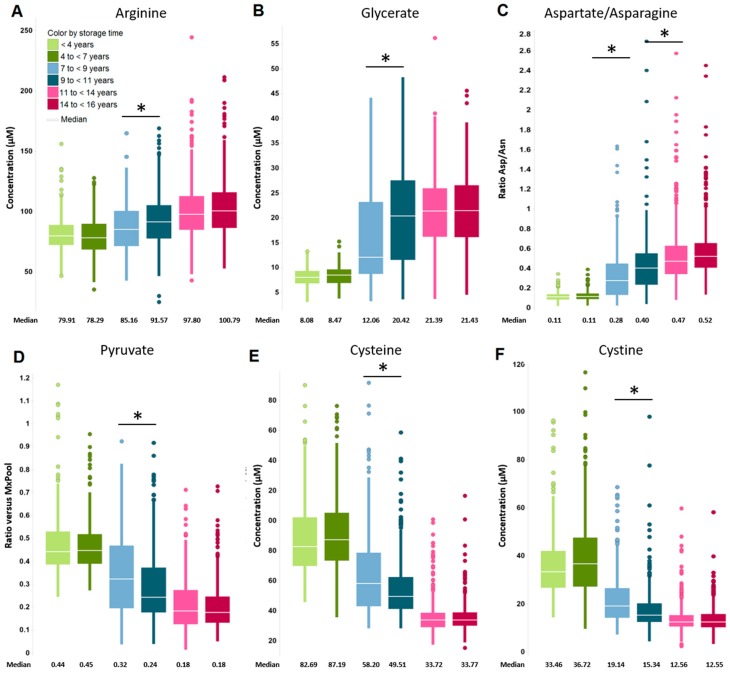
Metabolites or metabolite ratios that were elevated: (**A**) arginine, (**B**) glycerate, (**C**) the ratio of aspartate over asparagine or decreased (**D**) pyruvate, (**E**) cysteine, (**F**) cystine in concentration by prolonged sample storage at −80 °C. Asterisk denotes significant changes (*p* < 0.05, FDR < 0.05) within subject age groups. The legend is displayed in (**A**). Samples from one subject age group are shown in shades of the same color: 70 years of age = red; 75 years of age = blue; 80 years of age = green.

**Figure 2:**
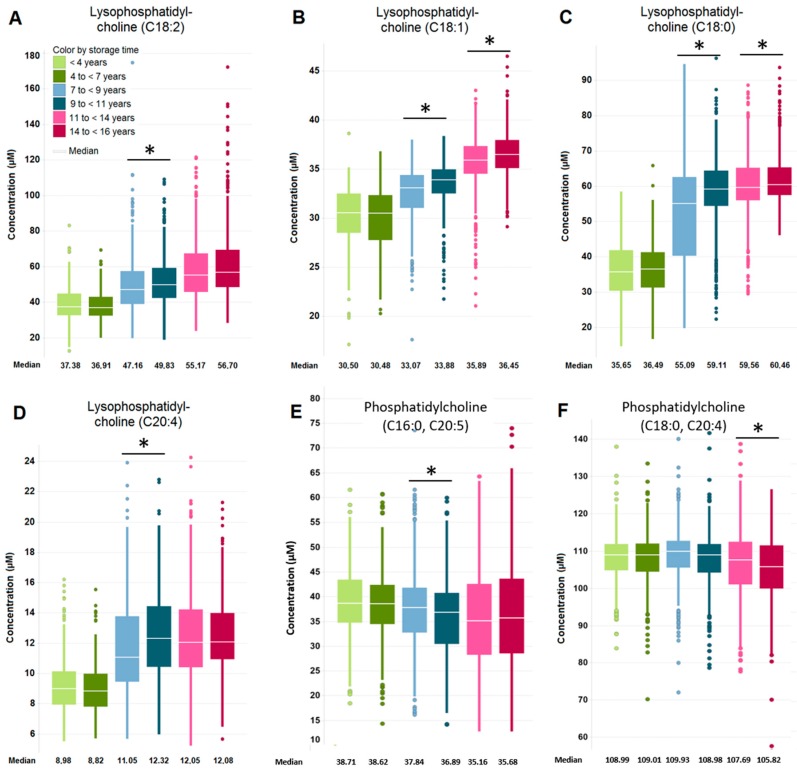
The effect of long-term storage at −80 °C on selected LPCs and polyunsaturated fatty acids containing PCs represented by box plots. (**A**) LPC (**C** 18:2), (**B**) LPC (**C** 18:1), (**C**) LPC (**C** 18:0), (**D**) LPC (**C** 20:4), (**E**) PC (**C** 16:0, **C** 20:5), (**F**) PC (**C** 18:0, **C** 20:4). Asterisk denotes significant changes (*p* < 0.05, FDR < 0.05) within subject age groups. The legend is displayed in (**A**). Samples from one subject age group are shown in shades of the same color: 70 years of age = red; 75 years of age = blue; 80 years of age = green.

**Figure 3:**
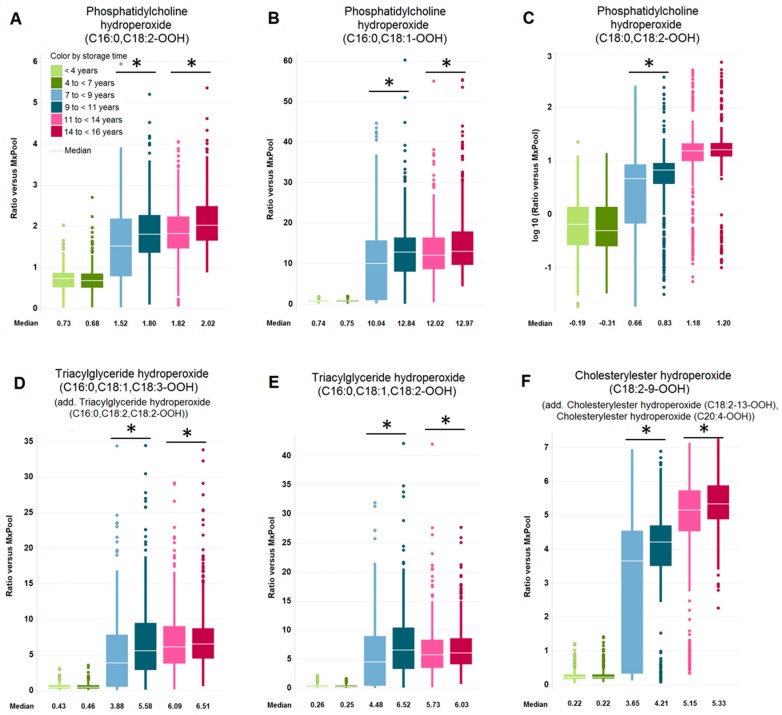
Long-term stability of PC hydroperoxides, TAG hydroperoxides, and CE hydroperoxide during storage at −80°C, shown by box plots. (**A**) PC hydroperoxide (**C** 16:0, **C** 18:2-OOH), (**B**) PC hydroperoxide (**C** 16:0, **C** 18:1-OOH), (**C**) PC hydroperoxide (**C** 18:0, **C** 18:2-OOH), (**D**) TAG hydroperoxide (**C** 16:0, C 18:1, **C** 18:3-OOH), (**E**) TAG hydroperoxide (**C** 16:0, **C** 18:1, **C** 18:2-OOH), (**F**) CE hydroperoxide (**C** 18:2-9-OOH). Asterisk denotes significant changes (*p* < 0.05, FDR < 0.05) within subject age groups. The legend is shown in (**A**). Samples from one subject age group are shown in shades of the same color: 70 years of age = red; 75 years of age = blue; 80 years of age = green. The term "add." (in addition) indicates that quantification could be disturbed by small amounts of the depicted metabolites that have identical analytical characteristics with respect to the quantification method.

Box plots were created to reveal the effects of prolonged storage at −80 °C on selected metabolites (Figure 1 Figure 2 Figure 3). Please note that the color coding in Figure 1, Figure 2 and Figure 3 represents the different subject age groups (shades of red: 70 years; shades of blue: 75 years; shades of green: 80 years). Levels of arginine and glycerate (Figure 1A,B) were significantly elevated after long-term storage. Interestingly, while the concentration of asparagine decreases upon long-term storage that of aspartate increases (Table S1) and hence the ratio of aspartate over asparagine increases over time (Figure 1C). 

In contrast, the levels of pyruvate and the amino acids cysteine and cystine were reduced over the analyzed storage period (Figure 1D–F). It is noteworthy, however, that most of the observed changes are significant (p < 0.05 and FDR < 0.05) only within the subject age group of samples stored for seven up to nine and those stored for nine up to eleven years (Table S1). Figure 2 shows the box plots of lysophosphatidylcholines (LPCs) C 18:2 (Figure 2A), C 18:1 (Figure 2B), C 18:0 (Figure 2C), and C 20:4 (Figure 2D). The amounts of these were increased by prolonged storage time within the subject age groups, while the levels of several phosphatidylcholines (PCs), in particular those containing polyunsaturated fatty acids (Figure 2E, F and Table S1) were reduced upon longer storage within the subject age groups. 

Figure 3 displays box plots of several lipid hydroperoxides, namely PC hydroperoxides (Figure 3A–C), triacylglyceride (TAG) hydroperoxides (Figure 3D, E), and a cholesterylester (Figure 3C, E) hydroperoxide (Figure 3F), that were all affected by longer storage within subject age groups and showed increased concentrations over time. Interestingly, in the case of lipid hydroperoxides the increased concentration (*p* < 0.05 and FDR < 0.05) with respect to longer storage time within subject age group was significant in most sample groups stored for more than 7 years.

Orthogonal projections to latent structures (OPLS) analysis within subject age groups revealed that all calculated multivariate OPLS models were poor predictors of storage time, i.e., their Q^2^cum values were <0.5 (Table S2). Furthermore, the total amount of variation in storage time that could be explained by the models was less than 50% in each model (R2Y(cum), Table S2). Interestingly though, when considering the contribution of metabolites to the separation according to storage time many metabolites that were found significantly altered in the univariate analysis were also prominent in the OPLS loadings (Figure S1) for models 1 and 2 (subject age groups 70 and 75 years, respectively).

### 2.3. Sample Quality is Affected by Long-Term Storage

**Figure 4:**
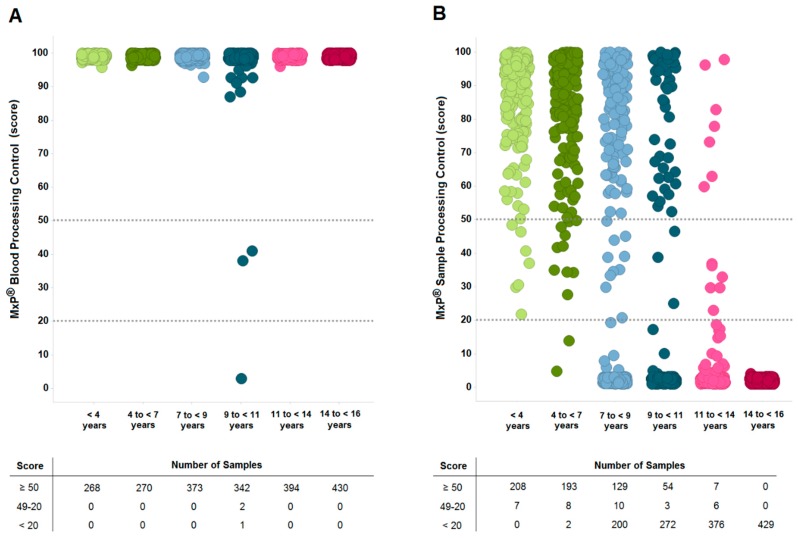
Sample quality assessment by the MxP Biofluids Quality Control assay: (**A**) MxP Blood Processing Control and (**B**) MxP Sample Processing Control were calculated in score for each sample, and the cut-offs were set to ≥50 for high-quality samples and <20 for low-quality samples. Please note that quality scores are not displayed for all samples as metabolite levels needed for the assay were in some cases below the limit of detection of the analytical method.

To further validate that the observed differences in metabolite concentrations are related to long-term storage effects, sample quality was evaluated using the MxP^®^ Biofluids Quality Control assay (Metanomics Health GmbH, Berlin, Germany). This assay provides two results: (i) MxP Blood Processing Control, which describes sample quality in terms of blood processing, and (ii) MxP Sample Processing Control, which shows sample quality with respect to plasma processing and sample storage. Scores of ≥50 are indicative for high-quality samples and scores of ≤20 for low-quality samples. Figure 4A shows that almost all samples had high MxP Blood Processing Control scores, indicating the high quality of blood collection and processing. In contrast, we found that MxP Sample Processing Control scores decreased over the storage period (Figure 4B), i.e., 97% of the samples stored for less than four years showed high-quality scores, while 100% of the samples stored between 14 and 16 years had low-quality scores. 

These results indicate that prolonged storage at −80 °C affects sample quality, and that the observed changes in metabolite concentrations are a result of storage time effects and not due to inadequate sample processing. Although it was documented that the EDTA plasma samples were stored continuously at −80 °C, it should be mentioned that generally reductions in quality scores could also be due to elevated temperatures during storage or transport of samples as well as prolonged blood or plasma processing times.

## 3. Discussion

In this work, we investigated the stability of 231 human plasma metabolites from ten different ontology classes during long-term storage at −80 °C over a period of up to 16 years. Due to the longitudinal study design with repeated sampling of the same individuals at 70, 75 and 80 years of age it was not possible to distinguish subject age-related from storage-time-dependent effects when comparing storage times across different subject age groups. Therefore, within each subject age group in this longitudinal study we investigated the influence of longer storage against shorter one. Hence the difference between the oldest and youngest samples in any one subject age group was about four to seven years. We found that 98% of the analyzed metabolites remained stable in the first seven years of storage, but upon prolonged storage of up to 16 years, time differences of few years resulted in a statistically significant change in concentration in up to 26% of the analyzed metabolites. All metabolite classes were affected to a certain degree, with complex lipids, fatty acids, energy metabolism molecules, and amino acids being the most affected by long-term storage. Therefore, these data indicate that the human plasma metabolome is adequately stable to long-term storage effects at −80 °C for up to seven years but is sensitive to even few years of additional storage if stored for longer time periods at −80 °C. Liquid nitrogen may be an alternative for highly unstable metabolites that require storage at lower temperatures but should be tested in further studies.

It is noteworthy, however, that many affected metabolites displayed significant changes only in the age group of 75 years, i.e., samples were stored for seven up to nine years and nine up to eleven years. Those significant effects vanished upon even longer storage of 14 up to 16 years versus eleven up to 14 years in the age group of 70 years.

Since the reasons for the observed effects may be related to oxidation reactions, acid-base-driven hydrolysis and enzymatic activities it is possible that these reactions reach a substrate-product-equilibrium upon prolonged storage of eleven up to 16 years. Previous studies have reported that hydrolysis cannot be stopped by an increase in viscosity, because the movement of H+ and OH− ions is possible even in solid ice [36,37]. In addition, freezing of aqueous solutions results in an increase in concentration of reactants, catalysts, electrolytes, and solvents in the remaining liquids, accompanied by changes in pH, solubility, viscosity, ionic strength, and thermodynamic properties [38].

However, the majority of samples from these storage groups were classified as poor quality according to the MxP Sample Processing Control meaning that irrespective of the decline of significant metabolite changes as compared to shorter storage times these samples should not be used in metabolomic analyses.

In a recent study, Haid et al. showed that amino acids are sensitive to long-term storage at −80 °C [35]. We confirmed this aspect for sample storage and further found that asparagine, cysteine and cystine, which are not included in their work, were all significantly reduced in concentration during storage. In contrast to the amino acids mentioned above, aspartate was significantly increased upon longer storage. In general, these effects can be explained by the fact that peptide bonds and amino acid side chains are susceptible to non-enzymatic hydrolysis. Particularly, asparagine can be converted to its dicarboxylic acid counterpart aspartate by deamination [39]. Cysteine instability can be explained by rapid oxidation to cystine. However, since cystine levels were also reduced over the storage period, the reduction of both cysteine and cystine could be a result of oxidative conversion to unidentified derivates, as described previously [33]. Moreover, non-enzymatic oxidation may explain the observed reduction of pyruvate concomitant with an increase in lactate (see Table S1) if samples were stored for up to eleven years versus those stored for up to nine years. Likewise, the observed increase in glycerate may derive from oxidation of glyceraldehyde, an intermediate of sugar metabolism. However, changes in pyruvate and lactate have also been noted upon prolonged blood or plasma processing and attributed to erythrocyte-derived enzymatic actions [14,40].

In addition to amino acids Haid et al. analyzed the alteration of LPCs and PCs after storage for five years. However, contrary to our observations LPCs were found reduced or unchanged in this publication with the PCs showing a more heterogenous behavior [35]. We could confirm that upon storage for up to seven years LPCs remained largely unchanged. However, after longer storage even four or five years of additional storage led to an increase in most of the measured LPCs. That is in good agreement with Kamlage et al., who found increased LPC concentrations upon prolonged serum processing at room temperature [18]. In contrast, the changes observed in PCs upon longer storage were inconsistent. Whereas, PCs with fatty acids of less than 20 carbon atoms and two double bonds at most remained mostly unchanged, PCs containing polyunsaturated fatty acids were reduced upon longer storage (see Table S1). Interestingly, we measured two arachidonic acid containing PCs. One of those is shown in Figure 2E. The other one, PC (C16:0, C20:4) (data not shown), displayed a similar behavior but failed to reach FDR < 0.05. The increase of LPCs and the concomitant decrease in polyunsaturated fatty acid-containing PCs may likely derive from phospholipase activity. Several isozymes of phospholipase A2 are calcium-independent or require minimal calcium amounts [41,42] and hence may be active in EDTA plasma even at low temperatures. Interestingly, several phospholipase A2 isozymes display great specificity for arachidonic acid at the sn-2 position [41,43] and may explain the reduction in arachidonic acid containing phosphatidylcholines. Furthermore, some phospholipase A2 enzymes are predominantly active on oxidized phosphatidylcholines that may derive from oxidation processes upon long-term storage [42]. It remains to be analyzed, however, if those polyunsaturated fatty acids are indeed released and if there are downstream reactions that may conceal their potentially increased free fatty acid concentrations. Because of their numerous double bonds, they are vulnerable to chemical reactions such as oxidation processes. The observed reduction of polyunsaturated fatty acid containing PCs is of great importance since arachidonic acid and its downstream products are important mediators of inflammatory processes [44] and hence studies aimed at detecting biomarkers in inflammatory diseases should always account for storage time as a potential confounder for their results.

Another interesting metabolite group that has been significantly impacted by long-term storage is lipid hydroperoxides, which have been increased over time by lipid oxidation and auto-oxidation processes. This so-called lipid peroxidation is a free radical-generating process that leads to the oxidative modification of lipids. Although lipid hydroperoxides have been described as biomarkers for the assessment the oxidative stress status and associated diseases [45], their susceptibility to long-term storage should be considered in the development of biomarkers containing these metabolites.

A limitation of our study is that lifestyle changes, diseases, etc. as a result of ageing of the individuals may also play a role on the changes of metabolite concentrations, i.e., effects of long-term storage on the plasma metabolome overlap with effects of biological ageing of the subjects. To overcome this problem metabolite concentrations were compared within subject age groups. However, that resulted in much smaller time differences between longer-stored and shorter-stored samples of seven, four and five years in the subject age groups of 70 years, 75 years, and 80 years, respectively. Furthermore, we focused our observations on EDTA plasma samples. Hence, it is possible that other blood-derived biospecimens such as serum or heparin plasma may display different changes or sensitivities in long-term storage.

In conclusion, access to high-quality samples that are collected and handled in standardized ways to minimize or even exclude confounding factors is key to the “bench to bedside” goal of translational research. We now have provided evidence that long-term storage of samples has a major impact on the stability of metabolites in human plasma, which in turn influences data analysis in metabolomics studies. In this context, we could demonstrate that metabolite profiling is well-suited for identifying low-quality samples prior to data analysis. However, since nearly all tested metabolites were stable for up to seven years at −80 °C, biomarker studies based on frozen samples should be performed as soon as possible after sampling.

## 4. Materials and Methods 

Study design. Human blood and plasma samples were collected from 70-year-old male and female individuals living in Uppsala, Sweden, between 2001 and 2004 for an epidemiological project known as the Prospective Investigation of the Vasculature in Uppsala Seniors study [46]. The subjects were randomly chosen from the register of the community of Uppsala, and 1016 out of 2025 invited people participated. Alive participants were resampled from 2006 to 2009 when they became 75 years old (n = 826), and from 2011 to 2014 when turning 80 years old (n = 602). Of these, 973, 824 and 601 subjects, respectively, were included in our present study. All blood samples were collected in the morning after an overnight fast, no smoking or medication was allowed after midnight. EDTA plasma was obtained as described elsewhere [47], and samples were stored in aliquots at −80 °C until further analysis. The study was conducted in adherence to the Declaration of Helsinki and was approved by the ethics committee of the Faculty of Medicine at Uppsala University. All participants gave their written informed consent prior to inclusion.

Metabolite profiling. Analysis of all EDTA plasma samples was performed between 2016 and 2017 by (i) gas chromatography-mass spectrometry (GC-MS) using an Agilent 6890 gas chromatograph coupled to an Agilent 5973 mass-selective detector and (ii) liquid chromatography-tandem mass spectrometry (LC-MS/MS) using an Agilent 1100 high-performance liquid chromatography system coupled to an Applied Biosystems API 4000 triple quadrupole mass spectrometer, as described in detail elsewhere [14,48,49]. Briefly, proteins were precipitated from plasma samples using three volumes of acetonitrile, and polar and nonpolar fractions were separated by adding water and a mixture of ethanol and dichloromethane (2:1, *v*/*v*). For GC-MS analysis, the nonpolar fraction was treated with methanol under acidic conditions to yield the fatty acid methyl esters derived from both free fatty acids and hydrolyzed complex lipids. The polar and nonpolar fractions were further derivatized with O-methyl-hydroxylamine hydrochloride to convert oxo-groups to O-methyl-oximes, and subsequently with a N-methyl-N-(trimethylsilyl)trifluoroacetamide prior to analysis. For LC-MS/MS analysis, both fractions were reconstituted in appropriate solvent mixtures, and high-performance liquid chromatography was performed by gradient elution using methanol/water/formic acid on reversed phase separation columns. Mass spectrometric detection technology was applied as described in patent WO2003073464 [50] which allows targeted and high-sensitivity multiple reaction monitoring (MRM) profiling in parallel to a full screen analysis. In brief, mass spectrometric detection was performed with repetitive cycles of MRM transitions for pre-selected metabolites followed by a full scan from a mass-to-charge ratio of 100 to 1000. The instrument was operated in positive ionization mode for metabolites in the nonpolar fraction, and in negative ionization mode for metabolites in the polar fraction. Metabolite identification was done by comparing sample data to authentic standards where applicable, as outlined previously [18].

Metabolite normalization and quantification. Metabolite profiling generated semi-quantitative data of metabolite concentrations calculated by determining metabolite levels in each study sample relative to metabolite concentrations in reference pool samples that were formed from aliquots of all study samples. To allow an experiment-comprehensive alignment of data sets, the semi-quantitative data were further normalized to the median of MxPool™ samples representing a pool of commercial human EDTA plasma containing more than 2000 different metabolites of known concentrations. A one-point calibration was used to quantify those metabolites that are present in the MxPool. Both types of pooled reference samples were run in parallel through the entire process.

Quality control. Quality control of the dataset comprised quality checks on peak, analyte, and sample level. Only those metabolites that met specific quality criteria as described in Meller et al. [51] were included in further statistical analyses. Furthermore, quality assessment of plasma samples was performed using the MxP Biofluids Quality Control assay. The metabolite panel and the applied algorithm are described in patent application WO2015145387A1 [52].

Statistical analysis and data visualization. Prior to statistical analysis, we applied a log10 transformation on the data for each metabolite to obtain an approximate normal distribution. The programs R (Version 3.4.4), Sartorius Stedim Biotech Simca (Version 15.0.2) and Tibco Spotfire (Version 6.0) were used for data analysis and visualization. Orthogonal projections to latent structures (OPLS) analysis was performed setting storage time in days as Y-variable. To enable read-out of common and differently regulated metabolites among the time points and to support the “Shared and Unique Structures” (SUS) plot, OPLS was done within the three respective subject age groups (70 years, 75 years, 80 years). Univariate statistical analysis was performed by analysis of variance (ANOVA) using a mixed linear model with “storage time group” as fixed and “subject” as random intercept (R package nlme). Similar to multivariate analysis the read out of all considered contrasts was within the three subject age groups (70 years, 75 years, 80 years) in order to prevent subject age confounding storage time effects. Hence, the considered contrasts comprised storage time groups four up to seven years versus less than four years (subject age group 80 years), nine up to eleven years versus seven up to nine years (subject age group 75 years) and 14 up to 16 years versus eleven up to 14 years (subject age group 70 years). The multiple test problem was addressed by calculating the false discovery rate (FDR) using the Benjamini and Hochberg method [53].

## Figures and Tables

**Table 1:** Number of significant metabolite changes (ANOVA; *p* < 0.05 and FDR < 0.05) due to different sample storage times at −80°C. Comparisons were made between storage times within subject age groups. Sample numbers per storage time group are: less than four years, n = 301; four to less than seven years, n = 300; seven to less than nine years, n = 425; nine to less than eleven years, n = 399; eleven to less than fourteen years, n = 460; and fourteen to less than sixteen years, n = 513.

	Significantly Changed Metabolites Long Storage vs. Shorter Storage within Subject Age Groups (Increase/Decrease). Inconsistencies in Percentage Sums are Due to Rounding.					
	Storage for 4 to <7 years vs. <4 years (subject age 80 years)		Storage for 9 to <11 years vs. 7 to <9 years (subject age 75 years)		Storage for 14 to <16 years vs. 11 to <14 years (subject age 70 years)	
Metabolite Ontology Class (Number of Metabolites)	Number	Percent Change	Number	Percent Change	Number	Percent Change
All (231)	5 (4/1)	2 (2/0)	59 (46/13)	26 (20/6)	27 (17/10)	12 (7/4)
Amino acids (22)	1 (1/0)	5 (5/0)	8 (4/4)	36 (18/18)	1 (1/0)	5 (5/0)
Amino acids related (15)	0	0	3 (2/1)	20 (13/7)	0	0
Carbohydrates and related (17)	0	0	2 (2/0)	12 (12/0)	2 (0/2)	12 (0/12)
Complex lipids, fatty acids and related (101)	0	0	27 (24/3)	27 (24/3)	15 (10/5)	15 (10/5)
Energy metabolism and related (17)	1 (1/0)	6 (6/0)	5 (3/2)	29 (17/12)	2 (1/1)	12 (6/6)
Miscellaneous (8)	1 (1/0)	13 (13/0)	2 (0/2)	25 (0/25)	0	0
Nucleobases and related (5)	0	0	0	0	1 (0/1)	20 (0/20)
Vitamins, cofactors and related (6)	1 (1/0)	17 (17/0)	1 (1/0)	17 (17/0)	0	0
Unknowns (38)	1 (0/1)	3 (0/3)	11 (10/1)	29 (26/3)	6 (5/1)	16 (13/3)

## Supplementary Materials

The following are available online at https://www.mdpi.com/2218-1989/9/5/99/s1. Table S1. Impact of different storage times at −80 °C on human plasma metabolite concentrations. Table S2. Results of OPLS models with storage time as Y-variable in different subject age groups. Figure S1. Shared and Unique Structures (SUS) plot comparing contribution of metabolites to the separation of samples according to storage time by OPLS.
