# Supplementary material for: Effects of Long-Term Storage at −80 °C on the Human Plasma Metabolome
Source: Metabolites. 2019 May 17;9(5):99. doi: 10.3390/metabo9050099 (PMC6572224; doi:10.3390/metabo9050099)
Supplement: Supplementary file 1 [file metabolites-09-00099-s001.pdf]

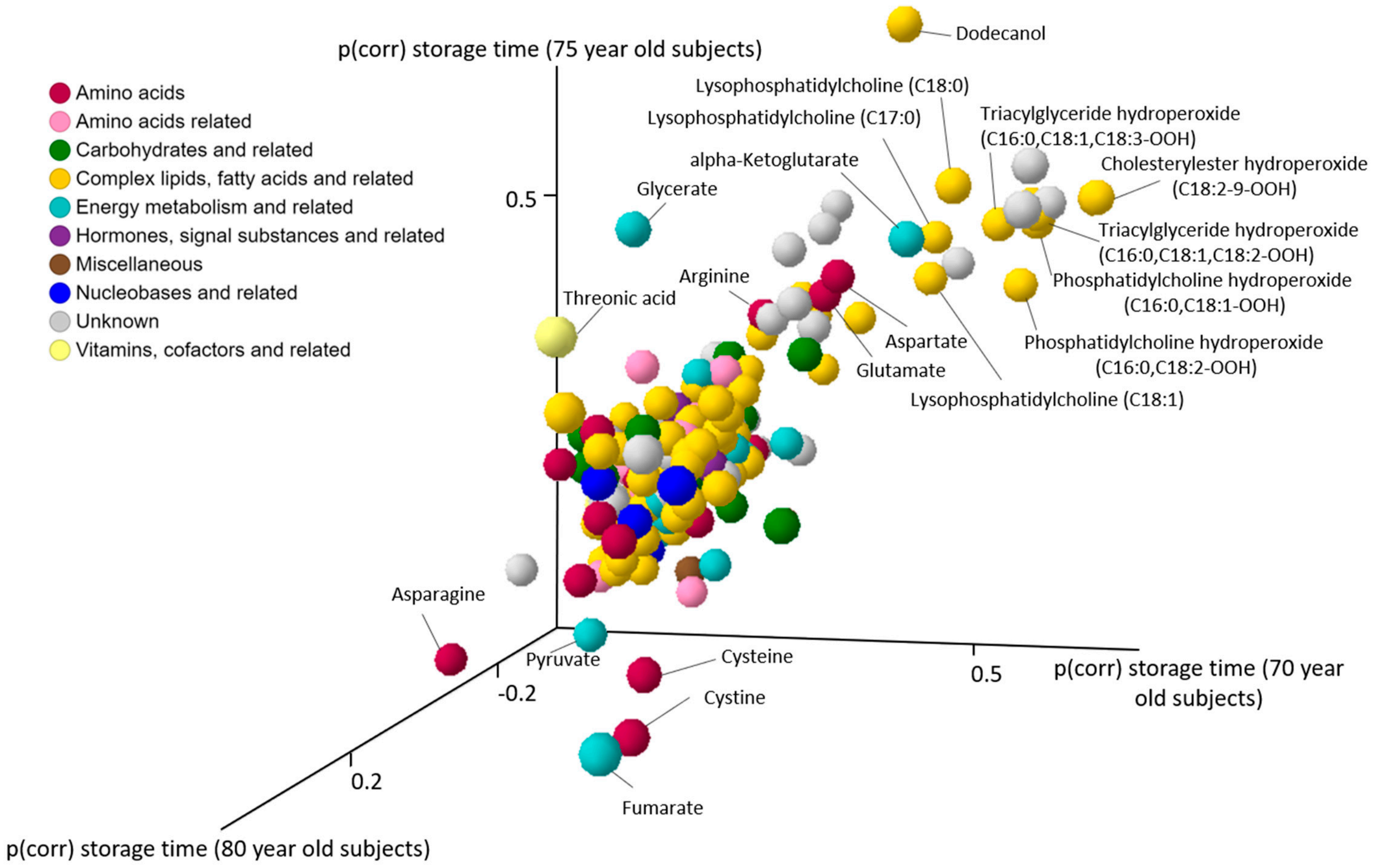

Figure 1. Shared and Unique Structures (SUS) plot comparing contribution of metabolites to the separation of samples according to storage time by OPLS. Multivariate Analysis was performed within the respective subject age groups. Metabolites are colored according to ontology class.

Table S1. Impact of different storage times at -80°C on human plasma metabolite concentrations. Fourteen analytes with final chemical structure identity pending are not indicated. Statistical analysis was performed by ANOVA. Metabolites with p-value < 0.05 and FDR < 0.05 were considered significantly altered (marked by * in the ratio column). The term "add." (in addition) indicates that quantification could be disturbed by small amounts of metabolites that have identical analytical characteristics with respect to the quantification method. Concentrations for Metabolites marked with (SQ) are semi-quantitative, i.e. ratio relative to MxPool.

Ontology 1	Metabolite	Storage for 4 to < 7 years, ratio vs. storage for < 4 years (subject age 80 years)	Storage for 9 to < 11 years, ratio vs. storage for 7 to < 9 years (subject age 75 years)	Storage for 14 to < 16 years, ratio vs. storage for 11 to < 14 years (subject age 70 years)	Storage for 4 to < 7 years, p-value vs. storage for < 4 years (subject age 80 years)	Storage for 9 to < 11 years, p-value vs. storage for 7 to < 9 years (subject age 75 years)	Storage for 14 to < 16 years, p-value vs. storage for 11 to < 14 years (subject age 70 years)	Storage for 4 to < 7 years vs. storage for < 4 years, FDR (subject age 80 years)	Storage for 9 to < 11 years vs. storage for 7 to < 9 years, FDR (subject age 75 years)	Storage for 14 to < 16 years vs. storage for 11 to < 14 years, FDR (subject age 70 years)	Storage for < 4 years, mean concentration [µmol/L] (subject age 80 years)	Storage for 4 to < 7 years, mean concentration [µmol/L] (subject age 80 years)	Storage for 7 to < 9 years, mean concentration [µmol/L] (subject age 75 years)	Storage for 9 to < 11 years, mean concentration [µmol/L] (subject age 75 years)	Storage for 11 to < 14 years, mean concentration [µmol/L] (subject age 70 years)	Storage for 14 to < 16 years, mean concentration [µmol/L] (subject age 70 years)
Amino acids	Aspartate	1.02	1.21*	1.07*	5.0E-01	5.2E-12	5.6E-03	9.5E-01	6.7E-11	4.8E-02	4.58	4.72	7.95	9.58	10.38	11.25
	Glutamate	1.03	1.35*	0.98	5.6E-01	3.9E-13	5.9E-01	9.5E-01	5.3E-12	8.3E-01	43.69	44.66	115.44	154.35	197.65	197.03
	Arginine	0.97	1.06*	1.03	4.8E-02	5.8E-05	4.0E-02	6.2E-01	3.6E-04	1.9E-01	79.98	77.81	84.25	89.80	97.18	100.68
	Asparagine	1.01	0.87*	0.96	6.1E-01	2.4E-10	3.1E-02	9.5E-01	2.6E-09	1.7E-01	42.26	41.86	32.25	27.71	22.78	21.53
	Glutamine	1.01	0.86*	1.01	7.3E-01	1.4E-07	8.1E-01	9.5E-01	1.2E-06	9.2E-01	670.72	667.03	472.35	404.03	381.75	381.57
	Histidine	1.01	1.03*	0.99	3.0E-01	1.0E-02	5.1E-01	9.5E-01	4.1E-02	7.8E-01	86.76	87.70	90.66	93.08	87.86	87.26
	Cysteine (add. Cystine)	1.03	0.88*	1.02	2.2E-01	3.3E-10	2.0E-01	9.5E-01	3.5E-09	5.0E-01	85.58	88.03	59.29	52.24	34.39	34.75
	Cystine	1.11*	0.82*	1.02	7.3E-04	1.7E-13	5.2E-01	2.8E-02	2.5E-12	7.8E-01	33.97	36.71	19.75	16.04	12.80	12.65
Amino acids related	5-Oxoproline (add. Folic acid, Glutamate, Glutamine) (SQ)	1.01	1.04*	1.00	4.7E-01	8.7E-05	8.7E-01	9.5E-01	5.0E-04	9.4E-01	1.10	1.10	1.18	1.22	1.25	1.26
	beta-Alanine (add. Pantothenic acid)	1.01	0.95*	1.01	6.8E-01	7.6E-03	4.5E-01	9.5E-01	3.3E-02	7.4E-01	1.84	1.85	1.86	1.75	1.52	1.52

	Ornithine (add. Arginine, Citrulline)	1.02	1.06*	0.99	2.0E-01	2.4E-06	5.0E-01	9.5E-01	1.7E-05	7.7E-01	57.13	57.82	63.75	67.44	59.01	58.80
Carbohydrates and related	3-Deoxyglucosone (SQ)	0.95	1.02	0.92*	1.6E-01	5.7E-01	3.1E-03	9.3E-01	7.2E-01	3.5E-02	0.98	0.93	0.93	0.95	0.99	0.91
	Ribose (add. Pentoses)	0.98	1.05*	0.98	2.7E-01	2.7E-03	2.3E-01	9.5E-01	1.3E-02	5.3E-01	0.34	0.33	0.33	0.35	0.35	0.34
	scyllo-Inositol	0.94	1.03	0.90*	1.9E-01	4.6E-01	1.2E-03	9.5E-01	6.1E-01	2.3E-02	2.19	2.14	1.95	2.03	1.87	1.76
	Erythronic acid	1.02	1.08*	1.02	2.3E-01	2.8E-06	2.4E-01	9.5E-01	1.8E-05	5.3E-01	3.09	3.15	3.39	3.66	3.26	3.34
Complex lipids, fatty acids and related	Ceramide (d18:1,C24:0)	1.02	1.07*	0.97	4.6E-01	9.5E-03	2.1E-01	9.5E-01	3.8E-02	5.2E-01	2.22	2.28	2.87	3.10	3.09	3.05
	Ceramide (d18:1,C24:1) (add. Ceramide (d18:2,C24:0))	0.99	1.08*	0.99	7.5E-01	2.2E-03	6.6E-01	9.5E-01	1.1E-02	8.7E-01	1.14	1.15	1.43	1.57	1.56	1.56
	Cholesterol, free	1.01	0.95*	1.01	4.6E-01	2.3E-03	4.9E-01	9.5E-01	1.1E-02	7.7E-01	912.56	920.18	752.58	716.77	698.28	702.03
	Isopalmitic acid (C16:0)	1.00	1.07*	1.00	9.1E-01	5.7E-03	8.8E-01	9.6E-01	2.5E-02	9.4E-01	3.28	3.26	3.20	3.44	3.37	3.40
	Eicosaenoic acid (C20:cis[11]1)	1.01	1.07*	1.00	6.0E-01	9.6E-03	8.5E-01	9.5E-01	3.9E-02	9.4E-01	14.68	14.94	15.71	16.88	16.30	16.48
	Palmitoleic acid (C16:cis[9]1)	1.05	1.11*	1.00	1.4E-01	1.3E-03	9.1E-01	9.3E-01	7.3E-03	9.4E-01	195.65	210.98	224.16	254.42	251.38	256.62
	conjugated Linoleic acid (C18:trans[9,11]2) (add. conjugated Linoleic acid (C18:cis[9]trans[11] 2))	1.01	1.08*	1.04	7.6E-01	2.2E-03	1.1E-01	9.5E-01	1.1E-02	3.6E-01	15.01	15.15	15.72	16.99	18.20	18.91
	Myristic acid (C14:0)	1.04	1.11*	1.02	2.9E-01	2.6E-03	4.8E-01	9.5E-01	1.3E-02	7.7E-01	100.66	106.29	113.93	127.52	121.82	125.88

Palmitic acid (C16:0)	1.02	1.06*	1.01	4.0E-01	8.4E-03	7.7E-01	9.5E-01	3.5E-02	9.2E-01	2456.42	2529.31	2762.15	2931.65	2720.84	2763.63
Dodecanol (SQ)	1.07	2.25*	1.17*	1.9E-01	2.2E-68	9.7E-05	9.5E-01	5.1E-66	3.4E-03	0.75	0.79	1.12	2.51	3.60	4.20
Cholesterylester hydroperoxide (C18:2-9-OOH) (add. Cholesterylester hydroperoxide (C18:2-13-OOH), Cholesterylester hydroperoxide (C20:4-OOH)) (SQ)	1.02	1.81*	1.24*	7.6E-01	6.7E-24	4.5E-05	9.5E-01	3.9E-22	2.2E-03	0.24	0.24	1.39	2.52	4.20	5.28
Phosphatidylcholine hydroperoxide (C16:0,C18:1-OOH) (SQ)	0.97	1.81*	1.28*	7.3E-01	2.1E-18	6.4E-05	9.5E-01	5.3E-17	2.7E-03	0.70	0.68	4.39	7.92	10.31	13.29
Phosphatidylcholine hydroperoxide (C16:0,C18:2-OOH) (SQ)	1.02	1.27*	1.18*	7.4E-01	6.7E-09	1.4E-05	9.5E-01	6.8E-08	1.3E-03	0.58	0.59	1.26	1.61	1.71	2.02
Phosphatidylcholine hydroperoxide (C18:0,C18:2-OOH) (SQ)	0.92	1.62*	1.25	4.8E-01	1.6E-06	1.6E-02	9.5E-01	1.2E-05	1.1E-01	0.62	0.57	2.60	4.21	11.71	14.60
Triacylglyceride hydroperoxide (C16:0,C18:1,C18:2-OOH) (SQ)	1.05	2.16*	1.29*	5.8E-01	2.2E-20	7.4E-04	9.5E-01	7.4E-19	1.5E-02	0.24	0.26	1.90	4.10	4.55	5.98
Triacylglyceride hydroperoxide (C16:0,C18:1,C18:3-OOH) (add. Triacylglyceride hydroperoxide	1.04	1.86*	1.23*	6.1E-01	5.1E-17	2.2E-03	9.5E-01	9.8E-16	2.9E-02	0.40	0.42	2.16	4.03	5.04	6.28

	(C16:0,C18:2,C18:2-OOH)) (SQ)															
	Glycerol, polar fraction	0.99	1.12*	1.04	6.4E-01	2.9E-07	7.5E-02	9.5E-01	2.3E-06	3.0E-01	144.75	143.40	148.96	167.64	136.43	141.93
	Glycerol-3-phosphate, polar fraction	0.95	2.09*	1.18*	5.5E-01	8.0E-30	3.3E-03	9.5E-01	9.3E-28	3.5E-02	2.16	2.05	13.13	27.49	23.07	27.53
	Lysophosphatidylcholine (C17:0)	0.99	1.21*	1.05	5.8E-01	4.5E-19	1.5E-02	9.5E-01	1.3E-17	1.1E-01	2.05	2.02	3.25	3.94	4.32	4.59
	Lysophosphatidylcholine (C18:0)	1.03	1.13*	1.04*	8.8E-02	3.7E-16	3.6E-03	8.7E-01	6.2E-15	3.6E-02	35.04	36.06	49.98	56.35	59.08	61.78
	Lysophosphatidylcholine (C18:1)	0.99	1.03*	1.03*	4.2E-01	7.9E-07	1.9E-05	9.5E-01	5.7E-06	1.3E-03	29.92	29.75	32.26	33.30	35.38	36.34
	Lysophosphatidylcholine (C18:2)	0.98	1.05*	1.04	4.7E-01	8.2E-03	2.6E-02	9.5E-01	3.5E-02	1.6E-01	37.28	36.90	47.60	50.15	55.48	58.38
	Lysophosphatidylcholine (C20:4)	0.98	1.08*	1.01	2.7E-01	4.8E-07	6.7E-01	9.5E-01	3.7E-06	8.7E-01	9.06	8.86	11.22	12.18	12.02	12.22
	Lysophosphatidylethanolamine (C22:5)	0.99	0.97*	1.00	4.5E-01	1.3E-02	8.5E-01	9.5E-01	4.9E-02	9.4E-01	1.81	1.79	1.68	1.63	1.61	1.60
	Phosphatidylcholine (C16:0,C16:0)	1.01	1.03*	1.01	5.8E-01	5.0E-03	4.3E-01	9.5E-01	2.3E-02	7.3E-01	14.45	14.54	15.02	15.42	14.96	15.12
	Phosphatidylcholine (C16:0,C20:5)	0.99	0.95*	1.00	5.3E-01	5.2E-03	7.8E-01	9.5E-01	2.3E-02	9.2E-01	38.23	37.89	36.77	35.01	34.66	34.91
	Phosphatidylcholine (C18:0,C20:3) (add. Phosphatidylcholine (C20:1,C18:2), Phosphatidylcholine (C20:2,C18:1))	1.01	1.00	0.97*	6.8E-01	1.0E+00	3.7E-03	9.5E-01	1.0E+00	3.6E-02	38.34	38.51	39.81	39.81	38.11	36.83

	Phosphatidylcholine (C18:0,C20:4)	1.00	0.99	0.99*	7.3E-01	9.4E-02	2.6E-03	9.5E-01	2.4E-01	3.1E-02	108.10	107.69	108.49	107.50	106.42	104.88
	Phosphatidylcholine (C18:1,C18:2) (add. Phosphatidylcholine (C16:0,C20:3))	1.01	1.00	0.98*	3.7E-01	4.6E-01	3.1E-04	9.5E-01	6.1E-01	7.8E-03	167.44	168.27	167.63	168.24	170.45	167.66
	erythro-Dihydrosphingosine (d16:0)	0.98	1.04	1.11*	7.0E-01	3.1E-01	3.9E-03	9.5E-01	5.1E-01	3.6E-02	37.67	36.89	41.94	43.76	38.61	43.79
	Sphingomyelin (d18:1,C16:0)	0.99	1.04*	1.02	6.5E-01	3.8E-05	6.3E-02	9.5E-01	2.5E-04	2.8E-01	105.31	104.83	105.20	109.78	121.88	124.27
	TAG (C16:0,C18:1,C18:3) (add. TAG (C16:0,C18:2,C18:2), TAG (C16:1,C18:1,C18:2))	0.97	1.02	0.91*	4.8E-01	6.8E-01	4.4E-03	9.5E-01	7.8E-01	3.9E-02	53.29	51.37	52.74	53.14	43.37	38.86
	TAG (C18:2,C18:2)	0.96	0.99	0.90*	3.8E-01	8.4E-01	3.9E-03	9.5E-01	8.8E-01	3.6E-02	82.25	78.02	80.52	78.92	61.19	54.19
Energy metabolism and related	alpha-Ketoglutarate (SQ)	1.04	1.37*	1.03	4.0E-01	1.3E-16	4.3E-01	9.5E-01	2.4E-15	7.3E-01	0.76	0.79	1.63	2.24	2.13	2.21
	Fumarate	1.06*	0.96*	1.00	2.0E-06	4.7E-05	8.8E-01	1.1E-04	3.0E-04	9.4E-01	2.76	2.91	2.76	2.65	2.40	2.39
	2-Hydroxybutyrate	1.03	0.96	0.93*	3.0E-01	1.0E-01	1.8E-03	9.5E-01	2.6E-01	2.8E-02	48.45	50.14	48.12	46.05	55.35	51.20
	Dodecanoylcarnitine	1.01	1.00	1.09*	8.0E-01	9.9E-01	2.1E-03	9.5E-01	9.9E-01	2.9E-02	0.17	0.17	0.16	0.15	0.11	0.12
	Glycerate	1.02	1.30*	0.99	5.5E-01	1.3E-17	6.8E-01	9.5E-01	2.8E-16	8.8E-01	7.84	8.10	13.69	17.83	19.71	20.04
	Lactate	0.98	1.07*	0.95	4.1E-01	2.2E-03	1.5E-02	9.5E-01	1.1E-02	1.1E-01	1026.14	1010.17	1159.17	1249.86	1044.13	992.26
	Pyruvate (add. Phosphoenolpyruvate (PEP)) (SQ)	1.01	0.85*	1.01	8.4E-01	6.1E-07	8.4E-01	9.5E-01	4.5E-06	9.4E-01	0.46	0.46	0.30	0.25	0.18	0.18

Miscellaneous	Ethylenediaminetetraacetic acid (EDTA)	1.07*	0.96*	1.00	1.4E-10	7.8E-05	6.6E-01	1.0E-08	4.7E-04	8.7E-01	9054.24	9689.02	9829.88	9452.85	8417.62	8432.42
	Quinic acid (add. Chlorogenic acid (CGA)) (SQ)	0.96	0.87*	0.97	4.7E-01	3.6E-03	5.0E-01	9.5E-01	1.7E-02	7.7E-01	1.10	1.00	0.95	0.83	0.84	0.80
Nucleobases and related	Uridine	0.97	0.96	0.95*	1.2E-01	6.4E-02	1.8E-03	8.8E-01	1.9E-01	2.8E-02	4.00	3.86	4.10	3.95	4.17	3.94
Vitamins, cofactors and related	Threonic acid	1.11*	1.10*	0.95	5.0E-04	3.0E-04	3.2E-02	2.3E-02	1.7E-03	1.7E-01	3.36	3.75	5.06	5.58	5.72	5.51

Table 2. Results of OPLS models with storage time as Y-variable in different subject age groups.

Subject Age Group	R^2Y(cum)	Q^2(cum)
70 years	0.289	0.188
75 years	0.485	0.308
80 years	0.415	0.181
